# Xyloccensin E

**DOI:** 10.1107/S1600536810016582

**Published:** 2010-05-15

**Authors:** Chanin Sarigaputi, Thapong Teerawatananond, Somchai Pengpreecha, Nongnuj Muangsin, Khanitha Pudhom

**Affiliations:** a Program in Biotechnology, Faculty of Science, Chulalongkorn University, Bangkok 10330, Thailand; b Research Centre of Bioorganic Chemistry, Department of Chemistry, Faculty of Science, Chulalongkorn University, Bangkok 10330, Thailand

## Abstract

The title compound (also known as phragmalin triacetate), C_35_H_42_O_14_, is a phragmalin-type limonoid extracted from *X. rumphii*. The mol­ecule consists of eight rings with the orthoacetate group bridged at positions 1, 8 and 9. The two five-carbocyclic rings (*A*_1_ and *A*_2_) and the dioxolane ring (*G*) adopt a distorted envelope conformation. The 1,3-dioxane ring (*E*) exists in a chair conformation. The six-carbocyclic rings (*B* and *C*) exhibit a twisted-boat conformation. The lactone ring has a half-chair conformation and the furan ring is planar (r.m.s. deviation = 0.002 Å). Rings *A*_1_/*B*, *A*_2_/*B*, *B*/*C*, *C*/*D* and *C*/*G* are all *cis*-fused. The two acet­oxy groups attached to ring *B* and the furan ring attached to the lactone ring are in equatorial positions. The porous crystal packing exhibits voids of 688 Å^3^ and weak inter­molecular C—H⋯O inter­actions. The absolute configuration was assigned on the basis of literature data.

## Related literature

For background to the structures of limonoids and their activities, see: Koul *et al.* (2004 ▶); Cui *et al.* (2005 ▶); Pudhom *et al.* (2009 ▶). For related structures and the assignment of the absolute configuration, see: Wu *et al.* (2004 ▶); Fan *et al.* (2007 ▶). For puckering parameters, see: Cremer & Pople (1975 ▶). 
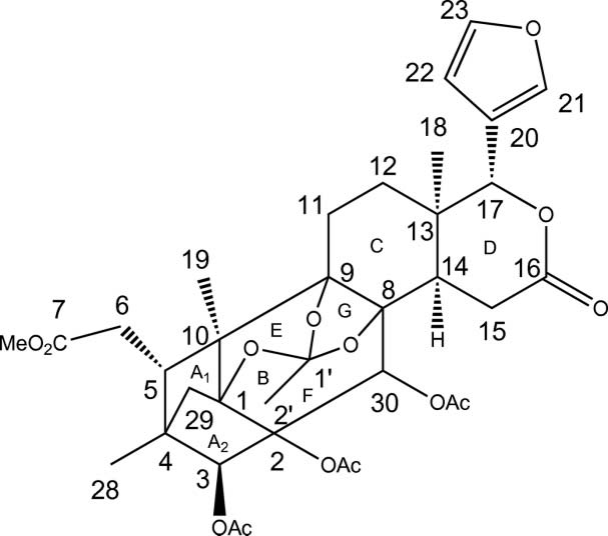


## Experimental

### 

#### Crystal data

C_35_H_42_O_14_
*M* *_r_* = 686.69
Hexagonal, 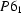

*a* = 17.7635 (5) Å
*c* = 19.6294 (6) Å
*V* = 5364.1 (3) Å^3^
*Z* = 6
Mo *K*α radiation
μ = 0.10 mm^−1^
*T* = 100 K
0.40 × 0.40 × 0.30 mm

#### Data collection

Bruker SMART APEXII CCD area-detector diffractometer
28307 measured reflections
4183 independent reflections
3738 reflections with *I* > 2σ(*I*)
*R*_int_ = 0.040

#### Refinement

*R*[*F* ^2^ > 2σ(*F* ^2^)] = 0.046
*wR*(*F* ^2^) = 0.130
*S* = 1.10
4183 reflections
445 parameters
1 restraint
H-atom parameters constrained
Δρ_max_ = 0.77 e Å^−3^
Δρ_min_ = −0.27 e Å^−3^

### 

Data collection: *APEX2* (Bruker, 2008 ▶); cell refinement: *SAINT* (Bruker, 2008 ▶); data reduction: *SAINT*; program(s) used to solve structure: *SHELXS97* (Sheldrick, 2008 ▶); program(s) used to refine structure: *SHELXL97* (Sheldrick, 2008 ▶); molecular graphics: *ORTEP-3* (Farrugia, 1997 ▶); software used to prepare material for publication: *publCIF* (Westrip, 2010 ▶).

## Supplementary Material

Crystal structure: contains datablocks global, I. DOI: 10.1107/S1600536810016582/cv2715sup1.cif

Structure factors: contains datablocks I. DOI: 10.1107/S1600536810016582/cv2715Isup2.hkl

Additional supplementary materials:  crystallographic information; 3D view; checkCIF report

## Figures and Tables

**Table 1:** Hydrogen-bond geometry (Å, °)

*D*—H⋯*A*	*D*—H	H⋯*A*	*D*⋯*A*	*D*—H⋯*A*
C6—H6*A*⋯O11^i^	0.97	2.41	3.326 (3)	157
C25—H25*B*⋯O1^ii^	0.96	2.34	3.294 (3)	176
C29—H29*A*⋯O10^iii^	0.97	2.51	3.348 (3)	145
C34—H34*A*⋯O11^iv^	0.96	2.62	3.293 (3)	127

Symmetry codes: (i) 
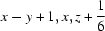
; (ii) 
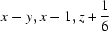
; (iii) 
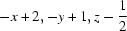
; (iv) 
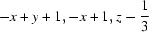
.

**Fig. 1.**
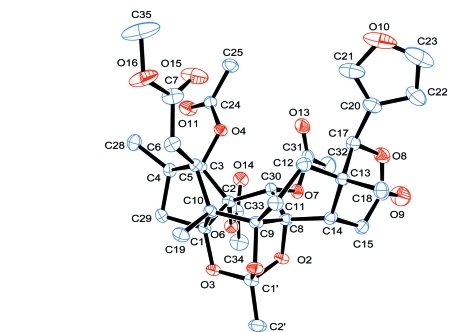
The molecular structure of the title compound, showing the atomic numbering scheme and 30% probability displacement ellipsoids. Hydrogen atoms are omitted for clarity.

**Fig. 2.**
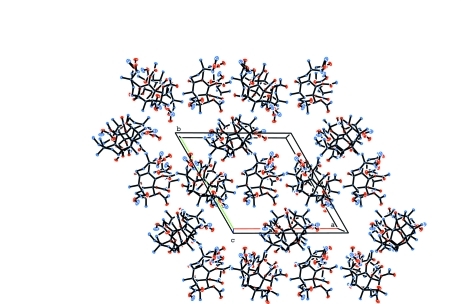
The crystal packing viewed along the c-axis and showing solvent accessible voids.

## supplementary crystallographic information

### Comment

Limonoid research from the Meliaceae family is of growing interest due to a range of biological activities, such as insect antifeedants and growth regulators, antibacterial, antifungal, antimalarial, anticancer and antiviral activities on humans (Koul *et al.*, 2004). Such a focused interest upon limonoids from the Meliaceae family has already resulted in a discovery of several limonoids with novel skeletons, mostly, but not exclusively, from the genus *Xylocarpus*, and, in particular, the cannonball mangrove, *Xylocarpus granatum* Koenig (Cui *et al.*, 2005; Pudhom *et al.*, 2009). Limonoid derivatives have been found in all *Xylocarpus* plants studied, but their distribution and content may vary both between different plant species, and between parts or geocultivars of the same species. This, combined with their wide ranging structural diversity and potential biological significance across this plant family, prompted us to investigate another plant in this genus, *Xylocarpus rumphii.* Herein, the complete assignments of NMR data and the crystal and molecular structure of Xyloccensin E obtained from the seeds of *X. rumphii *collected from Rayong Province, Thailand, in April 2009, were reported for the first time.

The molecule consists of eight rings with the orthoacetate group bridged at positions of 1, 8 and 9. The two five-carbocyclic rings A_1 _(C1, C2, C3, C4 and C29) and A_2_ (C1, C10, C5, C4 and C29) and the dioxlane ring G (C8, C9, O1, C1? and O2) adopt distorted envelope conformations [puckering parameters: *q_2_* = 0.639 Å, φ_2_ = -132.24° for A1, *q_2_* = 0.572 Å, φ_2_ = 164.56° for A_2_ and *q_2_* = 0.440 Å,φ_2_ = 31.13° for G]. The 1,3-dioxane ring E (C1?, C10, C9, O1, C1 and O3) exists as a chair conformation [puckering parameters: Q = 0.636 Å, q = 133.17° and φ = 17.29°]. The six carbocyclic rings B (C1, C2, C30, C8, C9 and C10) and C (C8, C9, C11, C12, C13 and C14) appear as twisted boat conformations [puckering parameters: Q = 0.815 Å, θ = -28.92°, φ = 84.96° and Q = 0.767 Å, θ = 90.57°, φ = -151.34°, respectively]. The lactone ring is a half-chair conformation and the furan ring is planar. Rings A_1_/B, A_2_/B, B/C, C/D, C/G are all cis-fused. The two acetoxy groups attached to ring B are in equatorial positions. The furan ring is attached to the lactone ring in equatorial position, and it is inclined at 56.8 (1) ° with respect to the lactone ring.

The structure is devoid of any classical hydrogen bonds. However, non-classical intra- and intermolecular (Table 1) hydrogen-bonding interactions of the type C—H···O are present in the structure. The crystal structure contains solvent accessible voids of 688 Å^3^.This might indicate that the crystal lost its solvent molecules of crystallization without collapsing of the structure.

### Experimental

Air-dried powdered seeds of *X. rumphii* (0.5 kg) were extracted with MeOH (2 L x 2, each for two days). The extract was concentrated under reduced pressure, followed by suspension in water and extraction with EtOAc. The resulting EtOAc crude extract (4.10 g) was chromatographed on a silica gel column eluted with a gradient of acetone–n-hexane (from 1:9 to 1:1) to yield 12 fractions. Fraction 12 (1.67 g) was further purified by silica gel column chromatography eluting with a gradient system of MeOH-CH_2_Cl_2_ (from 2:98 to 5:95) and recrystallized from methanol to afford xyloccensin E (1, 79.0 mg).

Colorless crystals; mp 168-171 ^o^C; ^1^H NMR (400 MHz, CDCl_3_) d 7.51 (1*H*, s, H-21), 7.40 (1*H*, s, H-23), 6.44 (1*H*, s, H-22), 6.30 (1*H*, s, H-30), 5.54 (1*H*, s, H-17), 5.10 (1*H*, s, H-3), 3.69 (3*H*, s, 7-COO*CH_3_*), 3.28 (1*H*, d, *J* = 20.3 Hz, H-15a), 2.96 (1*H*, d, *J* = 8.5 Hz, H-5), 2.70 (1*H*, m, H-15 b), 2.47 (1*H*, m, H-6a), 2.25 (3*H*, s, 1-OCO*CH_3_*), 2.24 (1*H*, m, H-6 b), 2.15 (3*H*, s, 3-OCO*CH_3_*), 2.07 (1*H*, m, H-11a), 1.98 (1*H*, m, H-29a), 1.94 (3*H*, s, 30-OCO*CH_3_*), 1.67 (1*H*, m, H-29 b), 1.66 (3*H*, s, 2?-CH_3_), 1.65 (1*H*, m, H-11 b), 1.54 (1*H*, m, H-12a), 1.30 (1*H*, m, H-12 b), 1.14 (3*H*, s, 19-CH_3_), 1.06 (3*H*, s, 18-CH_3_), 0.89 (3*H*, s, 28-CH_3_); ^13^C NMR (100 MHz, CDCl_3_) d 172.7 (C, C-7), 170.4 (C, C-16), 170.2 (C, 1-O*CO*CH_3_ and 3-O*CO*CH_3_), 168.6 (C, 30-O*CO*CH_3_), 143.0 (CH, C-23), 140.8 (CH, C-21), 121.1 (C, C-20), 119.0 (C, C-1?), 109.7 (CH, C-22), 86.8 (C, C-1), 85.9 (C, C-8), 85.3 (C, C-9), 85.2 (C, C-2), 81.1 (CH, C-3), 78.6 (CH, C-17), 69.3 (CH, C-30), 46.2 (C, C-4), 45.7 (C, C-10), 43.1 (C, C-14), 40.2 (CH_2_, C-29), 35.5 (CH, C-5), 34.3 (C, C-13), 33.3 (CH_2_, C-6), 29.1 (CH_2_, C-12), 26.5 (CH_2_, C-15), 25.4 (CH_2_, C-11), 21.7 (CH_3_, 3-OCO*CH_3_*), 21.6 (CH_3_, 30-OCO*CH_3_*), 21.1 (CH_3_, 1-OCO*CH_3_*), 21.0 (CH_3_, C-2?), 19.9 (CH_3_, C-18), 16.5 (CH_3_, C-19), 14.6 (CH_3_, C-28).; HRESIMS *m/z* 709.2490 [M+Na]^+^ (calcd for C_35_H_42_O_14_Na, 709.2492).

### Refinement

All H atoms were geometrically positioned and treated as riding atoms with distances C—H = 0.96 Å (CH_3_), 0.97 Å (CH_2_), 0.93 Å (CH), and *U*iso(H) = 1.20 *U*eq(C) for methylene and aromatic, 1.50 *U*eq(C) for methyl. Hydrogen atoms bonded to C25 and C35 were treated as rotationally disordered between two orientations each in a ratio 1:1. The absolute structure could not be determined from the X-ray analysis, but it was known from earlier work on related compounds (*e.g. *Wu *et al.*, 2004 and Fan *et al.*, 2007). 3652 Friedel pairs were therefore merged before the final refinement. The crystal structure contained solvent accessible voids of 688 Å^3^, showed no electrons in the voids. This might indicate that the crystal lost its solvent of crystallization without collapsing of the structure. The highest residual electron density peak (0.77 e Å^-3^) and the deepest hole (-0.27 e Å^-3^)  were located 0.77 and 0.29 Å at O9 and C15, respectively.

### Crystal data

**Table:**

C_35_H_42_O_14_	*F*(000) = 2184
*M**_r_* = 686.69	*D*_x_ = 1.275 Mg m^−^^3^
Hexagonal, *P*6_1_	Melting point: 168 K
Hall symbol: P 61	Mo *K*α radiation, λ = 0.71073 Å
*a* = 17.7635 (5) Å	µ = 0.10 mm^−^^1^
*c* = 19.6294 (6) Å	*T* = 100 K
*V* = 5364.1 (3) Å^3^	Prism, colourless
*Z* = 6	0.40 × 0.40 × 0.30 mm

### Data collection

**Table:**

Bruker SMART APEXII CCD area-detector diffractometer	3738 reflections with *I* > 2σ(*I*)
Radiation source: Mo	*R*_int_ = 0.040
graphite	θ_max_ = 27.5°, θ_min_ = 2.5°
φ and ω scans	*h* = −23→22
28307 measured reflections	*k* = −22→21
4183 independent reflections	*l* = −22→24

### Refinement

**Table:**

Refinement on *F*^2^	1 restraint
Least-squares matrix: full	H-atom parameters constrained
*R*[*F*^2^ > 2σ(*F*^2^)] = 0.046	*w* = 1/[σ^2^(*F*_o_^2^) + (0.085*P*)^2^ + 0.919*P*] where *P* = (*F*_o_^2^ + 2*F*_c_^2^)/3
*wR*(*F*^2^) = 0.130	(Δ/σ)_max_ = 0.029
*S* = 1.10	Δρ_max_ = 0.77 e Å^−^^3^
4183 reflections	Δρ_min_ = −0.27 e Å^−^^3^
445 parameters	

### Special details

**Table:**

Geometry. All esds (except the esd in the dihedral angle between two l.s. planes) are estimated using the full covariance matrix. The cell esds are taken into account individually in the estimation of esds in distances, angles and torsion angles; correlations between esds in cell parameters are only used when they are defined by crystal symmetry. An approximate (isotropic) treatment of cell esds is used for estimating esds involving l.s. planes.

### Fractional atomic coordinates and isotropic or equivalent isotropic displacement parameters (Å^2^)

**Table:**

	*x*	*y*	*z*	*U*_iso_*/*U*_eq_	Occ. (<1)
C1'	1.16441 (19)	0.58265 (19)	0.76883 (15)	0.0217 (6)	
C1	1.06376 (17)	0.61547 (17)	0.81972 (14)	0.0175 (5)	
C2'	1.2166 (2)	0.5971 (2)	0.70457 (17)	0.0296 (7)	
H2'1	1.1781	0.576	0.6661	0.044*	
H2'2	1.2482	0.5665	0.7077	0.044*	
H2'3	1.2567	0.6581	0.699	0.044*	
C2	0.98030 (17)	0.52150 (17)	0.82913 (13)	0.0170 (5)	
C3	0.91974 (17)	0.54361 (17)	0.87095 (14)	0.0170 (5)	
H3	0.8741	0.5414	0.8416	0.02*	
C4	0.98104 (17)	0.63768 (18)	0.89457 (15)	0.0189 (5)	
C5	1.05140 (17)	0.63106 (18)	0.93977 (14)	0.0182 (5)	
H5	1.0243	0.5723	0.959	0.022*	
C6	1.08925 (19)	0.69610 (19)	0.99894 (15)	0.0237 (6)	
H6A	1.1462	0.7048	1.0104	0.028*	
H6B	1.0967	0.7515	0.9842	0.028*	
C7	1.0333 (2)	0.6669 (2)	1.06132 (17)	0.0307 (7)	
C8	1.09628 (18)	0.48402 (18)	0.85465 (14)	0.0186 (5)	
C9	1.15837 (17)	0.57860 (18)	0.88338 (14)	0.0186 (5)	
C10	1.11894 (17)	0.63904 (17)	0.88473 (14)	0.0169 (5)	
C11	1.20720 (18)	0.57911 (19)	0.94751 (15)	0.0212 (6)	
H11A	1.2226	0.6312	0.9737	0.025*	
H11B	1.2605	0.5804	0.9347	0.025*	
C12	1.15201 (19)	0.49887 (19)	0.99166 (15)	0.0220 (6)	
H12A	1.0969	0.4954	1.0017	0.026*	
H12B	1.1817	0.5046	1.0345	0.026*	
C13	1.13502 (19)	0.41414 (19)	0.95545 (15)	0.0211 (6)	
C14	1.13326 (18)	0.42580 (19)	0.87763 (15)	0.0201 (5)	
H14	1.1948	0.4589	0.8648	0.024*	
C15	1.0988 (2)	0.3398 (2)	0.83957 (15)	0.0247 (6)	
H15A	1.0761	0.3454	0.7961	0.03*	
H15B	1.1477	0.3314	0.83	0.03*	
C16	1.0297 (2)	0.2590 (2)	0.87376 (17)	0.0309 (7)	
C17	1.0464 (2)	0.33966 (19)	0.98038 (16)	0.0240 (6)	
H17	1.0036	0.3588	0.9758	0.029*	
C18	1.2087 (2)	0.3951 (2)	0.97206 (16)	0.0268 (6)	
H18A	1.1967	0.3416	0.9508	0.04*	
H18B	1.2124	0.3903	1.0205	0.04*	
H18C	1.2628	0.4416	0.9552	0.04*	
C19	1.19506 (18)	0.73425 (18)	0.88085 (16)	0.0230 (6)	
H19A	1.2293	0.7481	0.9216	0.035*	
H19B	1.1722	0.7728	0.8764	0.035*	
H19C	1.2308	0.7407	0.8421	0.035*	
C20	1.0459 (2)	0.3137 (2)	1.05295 (18)	0.0308 (7)	
C21	1.0287 (3)	0.3468 (3)	1.10853 (19)	0.0436 (9)	
H21	1.0145	0.3906	1.1076	0.052*	
C22	1.0637 (3)	0.2486 (3)	1.0784 (2)	0.0523 (11)	
H22	1.0777	0.2132	1.0528	0.063*	
C23	1.0561 (3)	0.2487 (4)	1.1461 (3)	0.0651 (15)	
H23	1.0645	0.2124	1.1754	0.078*	
C24	0.79901 (18)	0.46072 (18)	0.94503 (15)	0.0212 (5)	
C25	0.7784 (2)	0.4132 (2)	1.01126 (17)	0.0280 (6)	
H25A	0.828	0.4098	1.0265	0.042*	0.5
H25B	0.73	0.3555	1.0053	0.042*	0.5
H25C	0.764	0.4436	1.0445	0.042*	0.5
H25D	0.72	0.3961	1.0244	0.042*	0.5
H25E	0.8181	0.4504	1.0456	0.042*	0.5
H25F	0.784	0.3624	1.0064	0.042*	0.5
C28	0.9336 (2)	0.6798 (2)	0.92673 (17)	0.0257 (6)	
H28A	0.975	0.7379	0.9405	0.039*	
H28B	0.9018	0.6467	0.9658	0.039*	
H28C	0.8941	0.6814	0.8942	0.039*	
C29	1.02450 (19)	0.67419 (18)	0.82469 (15)	0.0205 (5)	
H29A	0.9828	0.6636	0.7888	0.025*	
H29B	1.0681	0.7354	0.8265	0.025*	
C30	1.00034 (17)	0.45462 (17)	0.86481 (14)	0.0171 (5)	
H30	0.9871	0.4515	0.9136	0.021*	
C31	0.87864 (19)	0.30753 (19)	0.86851 (19)	0.0278 (7)	
C32	0.8275 (2)	0.2305 (2)	0.8248 (2)	0.0422 (9)	
H32A	0.7737	0.1915	0.8469	0.063*	
H32B	0.86	0.2014	0.8171	0.063*	
H32C	0.8158	0.2487	0.7819	0.063*	
C33	0.86812 (19)	0.43362 (19)	0.74661 (16)	0.0224 (6)	
C34	0.8570 (2)	0.4182 (2)	0.67120 (18)	0.0360 (8)	
H34A	0.8444	0.3601	0.6613	0.054*	
H34B	0.9096	0.459	0.6484	0.054*	
H34C	0.8099	0.4256	0.6556	0.054*	
C35	1.0148 (3)	0.7155 (4)	1.1691 (2)	0.0732 (18)	
H35A	1.0391	0.7675	1.196	0.11*	0.5
H35B	1.0208	0.6716	1.1929	0.11*	0.5
H35C	0.9543	0.6952	1.161	0.11*	0.5
H35D	0.9704	0.6554	1.1706	0.11*	0.5
H35E	0.9887	0.7513	1.1737	0.11*	0.5
H35F	1.0552	0.7277	1.2057	0.11*	0.5
O1	1.21889 (12)	0.61294 (13)	0.82626 (11)	0.0214 (4)	
O2	1.10977 (13)	0.49352 (13)	0.78162 (10)	0.0208 (4)	
O3	1.11411 (13)	0.62397 (13)	0.76107 (10)	0.0207 (4)	
O4	0.88190 (12)	0.48674 (13)	0.92764 (10)	0.0184 (4)	
O6	0.95079 (12)	0.49548 (12)	0.75992 (10)	0.0198 (4)	
O7	0.94979 (13)	0.36999 (12)	0.83415 (11)	0.0215 (4)	
O8	1.01541 (16)	0.25960 (14)	0.94147 (12)	0.0308 (5)	
O9	0.9896 (2)	0.19177 (17)	0.84406 (16)	0.0500 (7)	
O10	1.0349 (2)	0.3077 (2)	1.16646 (14)	0.0602 (10)	
O11	0.75158 (15)	0.47641 (17)	0.91176 (13)	0.0351 (6)	
O13	0.86291 (15)	0.31476 (14)	0.92703 (13)	0.0318 (5)	
O14	0.81228 (14)	0.39672 (14)	0.78855 (12)	0.0266 (5)	
O15	0.97453 (19)	0.59611 (19)	1.07362 (14)	0.0504 (8)	
O16	1.05994 (17)	0.7337 (2)	1.10510 (14)	0.0500 (8)	

### Atomic displacement parameters (Å^2^)

**Table:**

	*U*^11^	*U*^22^	*U*^33^	*U*^12^	*U*^13^	*U*^23^
C1'	0.0198 (13)	0.0228 (14)	0.0195 (13)	0.0085 (11)	0.0022 (11)	0.0031 (11)
C1	0.0163 (12)	0.0178 (12)	0.0169 (12)	0.0073 (10)	0.0012 (10)	0.0031 (10)
C2'	0.0278 (16)	0.0324 (16)	0.0252 (16)	0.0126 (13)	0.0102 (13)	0.0070 (13)
C2	0.0157 (12)	0.0181 (12)	0.0145 (12)	0.0065 (10)	−0.0021 (9)	−0.0002 (10)
C3	0.0150 (12)	0.0178 (12)	0.0176 (12)	0.0077 (10)	0.0001 (10)	0.0019 (10)
C4	0.0143 (12)	0.0179 (13)	0.0226 (13)	0.0066 (10)	−0.0018 (10)	−0.0003 (10)
C5	0.0157 (12)	0.0185 (12)	0.0193 (13)	0.0076 (10)	−0.0009 (10)	0.0009 (10)
C6	0.0184 (13)	0.0243 (14)	0.0244 (14)	0.0077 (11)	−0.0026 (11)	−0.0056 (12)
C7	0.0228 (15)	0.0396 (18)	0.0222 (14)	0.0099 (14)	−0.0055 (12)	−0.0079 (13)
C8	0.0164 (12)	0.0224 (13)	0.0180 (13)	0.0104 (11)	0.0009 (10)	0.0022 (10)
C9	0.0124 (11)	0.0213 (13)	0.0205 (13)	0.0072 (10)	0.0016 (10)	0.0028 (10)
C10	0.0141 (12)	0.0165 (12)	0.0182 (12)	0.0063 (10)	0.0000 (9)	0.0013 (10)
C11	0.0182 (13)	0.0255 (14)	0.0193 (13)	0.0104 (11)	−0.0019 (10)	0.0008 (11)
C12	0.0206 (13)	0.0269 (14)	0.0194 (13)	0.0124 (11)	−0.0004 (11)	0.0013 (11)
C13	0.0216 (13)	0.0268 (14)	0.0184 (13)	0.0146 (12)	0.0034 (11)	0.0047 (11)
C14	0.0186 (13)	0.0263 (14)	0.0194 (13)	0.0142 (11)	0.0015 (10)	0.0018 (11)
C15	0.0297 (15)	0.0292 (15)	0.0214 (14)	0.0194 (13)	0.0013 (12)	0.0000 (11)
C16	0.0389 (18)	0.0290 (16)	0.0287 (16)	0.0200 (15)	0.0031 (14)	0.0008 (13)
C17	0.0251 (14)	0.0246 (14)	0.0230 (14)	0.0129 (12)	0.0006 (11)	0.0011 (11)
C18	0.0266 (15)	0.0343 (16)	0.0263 (15)	0.0204 (13)	−0.0008 (12)	0.0039 (13)
C19	0.0151 (12)	0.0179 (13)	0.0261 (14)	0.0008 (11)	0.0010 (11)	0.0021 (11)
C20	0.0243 (15)	0.0332 (17)	0.0287 (16)	0.0096 (13)	0.0038 (12)	0.0080 (13)
C21	0.0390 (19)	0.040 (2)	0.0275 (18)	0.0015 (16)	0.0070 (15)	0.0009 (15)
C22	0.064 (3)	0.062 (3)	0.042 (2)	0.040 (2)	0.010 (2)	0.024 (2)
C23	0.056 (3)	0.085 (4)	0.039 (2)	0.024 (3)	0.002 (2)	0.030 (2)
C24	0.0182 (13)	0.0213 (13)	0.0242 (14)	0.0099 (11)	0.0025 (11)	0.0020 (11)
C25	0.0231 (14)	0.0292 (16)	0.0284 (15)	0.0106 (13)	0.0066 (12)	0.0080 (12)
C28	0.0241 (14)	0.0270 (15)	0.0302 (16)	0.0159 (12)	−0.0019 (12)	−0.0015 (12)
C29	0.0213 (13)	0.0187 (13)	0.0214 (13)	0.0100 (11)	0.0000 (11)	0.0030 (11)
C30	0.0143 (12)	0.0154 (12)	0.0205 (13)	0.0066 (10)	−0.0010 (10)	0.0005 (10)
C31	0.0194 (14)	0.0179 (14)	0.0444 (19)	0.0079 (11)	0.0013 (13)	0.0037 (13)
C32	0.0288 (17)	0.0205 (15)	0.066 (3)	0.0035 (13)	0.0037 (17)	−0.0040 (16)
C33	0.0208 (13)	0.0195 (13)	0.0264 (14)	0.0097 (12)	−0.0066 (12)	−0.0034 (11)
C34	0.0328 (18)	0.0370 (18)	0.0273 (17)	0.0091 (15)	−0.0090 (14)	−0.0083 (13)
C35	0.039 (2)	0.111 (4)	0.036 (2)	0.012 (3)	0.0052 (18)	−0.035 (3)
O1	0.0151 (9)	0.0247 (10)	0.0207 (10)	0.0072 (8)	0.0027 (8)	0.0026 (8)
O2	0.0215 (10)	0.0227 (10)	0.0163 (9)	0.0095 (8)	0.0026 (7)	0.0038 (8)
O3	0.0208 (9)	0.0225 (10)	0.0171 (9)	0.0094 (8)	0.0036 (7)	0.0035 (8)
O4	0.0142 (9)	0.0197 (9)	0.0200 (10)	0.0075 (7)	0.0012 (7)	0.0045 (7)
O6	0.0195 (9)	0.0199 (9)	0.0175 (9)	0.0080 (8)	−0.0043 (7)	−0.0023 (7)
O7	0.0188 (9)	0.0160 (9)	0.0276 (10)	0.0072 (8)	−0.0006 (8)	−0.0003 (8)
O8	0.0372 (12)	0.0228 (10)	0.0284 (11)	0.0119 (10)	0.0035 (10)	0.0018 (9)
O9	0.0683 (19)	0.0263 (13)	0.0443 (15)	0.0154 (13)	0.0063 (14)	−0.0075 (11)
O10	0.0501 (17)	0.068 (2)	0.0228 (14)	0.0001 (16)	0.0038 (12)	0.0066 (14)
O11	0.0226 (11)	0.0506 (14)	0.0375 (13)	0.0225 (11)	0.0073 (10)	0.0155 (11)
O13	0.0267 (11)	0.0232 (11)	0.0425 (14)	0.0103 (9)	0.0095 (10)	0.0090 (10)
O14	0.0207 (10)	0.0248 (10)	0.0294 (11)	0.0078 (9)	−0.0025 (9)	−0.0004 (9)
O15	0.0473 (16)	0.0454 (16)	0.0280 (13)	0.0003 (13)	0.0066 (12)	0.0006 (11)
O16	0.0336 (13)	0.0577 (17)	0.0362 (15)	0.0059 (12)	0.0084 (11)	−0.0241 (13)

### Geometric parameters (Å, °)

**Table:**

C1'—O2	1.405 (3)	C17—O8	1.458 (4)
C1'—O1	1.406 (4)	C17—C20	1.496 (4)
C1'—O3	1.420 (4)	C17—H17	0.98
C1'—C2'	1.509 (4)	C18—H18A	0.96
C1—O3	1.419 (3)	C18—H18B	0.96
C1—C29	1.520 (4)	C18—H18C	0.96
C1—C10	1.534 (4)	C19—H19A	0.96
C1—C2	1.595 (4)	C19—H19B	0.96
C2'—H2'1	0.96	C19—H19C	0.96
C2'—H2'2	0.96	C20—C21	1.346 (6)
C2'—H2'3	0.96	C20—C22	1.431 (5)
C2—O6	1.446 (3)	C21—O10	1.365 (5)
C2—C3	1.552 (4)	C21—H21	0.93
C2—C30	1.566 (4)	C22—C23	1.336 (7)
C3—O4	1.425 (3)	C22—H22	0.93
C3—C4	1.541 (4)	C23—O10	1.342 (8)
C3—H3	0.98	C23—H23	0.93
C4—C28	1.515 (4)	C24—O11	1.204 (4)
C4—C29	1.548 (4)	C24—O4	1.348 (3)
C4—C5	1.584 (4)	C24—C25	1.493 (4)
C5—C6	1.536 (4)	C25—H25A	0.96
C5—C10	1.567 (4)	C25—H25B	0.96
C5—H5	0.98	C25—H25C	0.96
C6—C7	1.497 (4)	C25—H25D	0.96
C6—H6A	0.97	C25—H25E	0.96
C6—H6B	0.97	C25—H25F	0.96
C7—O15	1.190 (4)	C28—H28A	0.96
C7—O16	1.345 (4)	C28—H28B	0.96
C8—O2	1.449 (3)	C28—H28C	0.96
C8—C30	1.525 (4)	C29—H29A	0.97
C8—C14	1.544 (4)	C29—H29B	0.97
C8—C9	1.582 (4)	C30—O7	1.442 (3)
C9—O1	1.459 (3)	C30—H30	0.98
C9—C11	1.526 (4)	C31—O13	1.204 (4)
C9—C10	1.548 (4)	C31—O7	1.372 (4)
C10—C19	1.552 (4)	C31—C32	1.481 (5)
C11—C12	1.532 (4)	C32—H32A	0.96
C11—H11A	0.97	C32—H32B	0.96
C11—H11B	0.97	C32—H32C	0.96
C12—C13	1.551 (4)	C33—O14	1.201 (4)
C12—H12A	0.97	C33—O6	1.348 (3)
C12—H12B	0.97	C33—C34	1.500 (5)
C13—C18	1.541 (4)	C34—H34A	0.96
C13—C14	1.544 (4)	C34—H34B	0.96
C13—C17	1.544 (4)	C34—H34C	0.96
C14—C15	1.527 (4)	C35—O16	1.437 (5)
C14—H14	0.98	C35—H35A	0.96
C15—C16	1.502 (5)	C35—H35B	0.96
C15—H15A	0.97	C35—H35C	0.96
C15—H15B	0.97	C35—H35D	0.96
C16—O9	1.193 (4)	C35—H35E	0.96
C16—O8	1.354 (4)	C35—H35F	0.96
			
O2—C1'—O1	104.1 (2)	C13—C18—H18C	109.5
O2—C1'—O3	110.0 (2)	H18A—C18—H18C	109.5
O1—C1'—O3	112.3 (2)	H18B—C18—H18C	109.5
O2—C1'—C2'	111.2 (2)	C10—C19—H19A	109.5
O1—C1'—C2'	111.0 (2)	C10—C19—H19B	109.5
O3—C1'—C2'	108.3 (2)	H19A—C19—H19B	109.5
O3—C1—C29	117.4 (2)	C10—C19—H19C	109.5
O3—C1—C10	111.3 (2)	H19A—C19—H19C	109.5
C29—C1—C10	102.1 (2)	H19B—C19—H19C	109.5
O3—C1—C2	114.3 (2)	C21—C20—C22	105.1 (4)
C29—C1—C2	101.9 (2)	C21—C20—C17	127.2 (3)
C10—C1—C2	108.8 (2)	C22—C20—C17	127.7 (3)
C1'—C2'—H2'1	109.5	C20—C21—O10	111.1 (4)
C1'—C2'—H2'2	109.5	C20—C21—H21	124.5
H2'1—C2'—H2'2	109.5	O10—C21—H21	124.5
C1'—C2'—H2'3	109.5	C23—C22—C20	106.5 (5)
H2'1—C2'—H2'3	109.5	C23—C22—H22	126.8
H2'2—C2'—H2'3	109.5	C20—C22—H22	126.8
O6—C2—C3	113.0 (2)	C22—C23—O10	111.4 (4)
O6—C2—C30	111.2 (2)	C22—C23—H23	124.3
C3—C2—C30	113.9 (2)	O10—C23—H23	124.3
O6—C2—C1	103.0 (2)	O11—C24—O4	123.7 (3)
C3—C2—C1	101.3 (2)	O11—C24—C25	127.0 (3)
C30—C2—C1	113.6 (2)	O4—C24—C25	109.3 (2)
O4—C3—C4	111.2 (2)	C24—C25—H25A	109.5
O4—C3—C2	112.0 (2)	C24—C25—H25B	109.5
C4—C3—C2	103.3 (2)	H25A—C25—H25B	109.5
O4—C3—H3	110.1	C24—C25—H25C	109.5
C4—C3—H3	110.1	H25A—C25—H25C	109.5
C2—C3—H3	110.1	H25B—C25—H25C	109.5
C28—C4—C3	113.3 (2)	C24—C25—H25D	109.5
C28—C4—C29	116.5 (2)	H25A—C25—H25D	141.1
C3—C4—C29	97.0 (2)	H25B—C25—H25D	56.3
C28—C4—C5	117.2 (2)	H25C—C25—H25D	56.3
C3—C4—C5	104.1 (2)	C24—C25—H25E	109.5
C29—C4—C5	106.3 (2)	H25A—C25—H25E	56.3
C6—C5—C10	115.2 (2)	H25B—C25—H25E	141.1
C6—C5—C4	115.6 (2)	H25C—C25—H25E	56.3
C10—C5—C4	101.8 (2)	H25D—C25—H25E	109.5
C6—C5—H5	107.9	C24—C25—H25F	109.5
C10—C5—H5	107.9	H25A—C25—H25F	56.3
C4—C5—H5	107.9	H25B—C25—H25F	56.3
C7—C6—C5	113.1 (2)	H25C—C25—H25F	141.1
C7—C6—H6A	109	H25D—C25—H25F	109.5
C5—C6—H6A	109	H25E—C25—H25F	109.5
C7—C6—H6B	109	C4—C28—H28A	109.5
C5—C6—H6B	109	C4—C28—H28B	109.5
H6A—C6—H6B	107.8	H28A—C28—H28B	109.5
O15—C7—O16	122.6 (3)	C4—C28—H28C	109.5
O15—C7—C6	127.7 (3)	H28A—C28—H28C	109.5
O16—C7—C6	109.7 (3)	H28B—C28—H28C	109.5
O2—C8—C30	105.1 (2)	C1—C29—C4	94.2 (2)
O2—C8—C14	105.4 (2)	C1—C29—H29A	112.9
C30—C8—C14	120.6 (2)	C4—C29—H29A	112.9
O2—C8—C9	104.0 (2)	C1—C29—H29B	112.9
C30—C8—C9	112.5 (2)	C4—C29—H29B	112.9
C14—C8—C9	107.6 (2)	H29A—C29—H29B	110.3
O1—C9—C11	109.5 (2)	O7—C30—C8	108.0 (2)
O1—C9—C10	102.6 (2)	O7—C30—C2	110.2 (2)
C11—C9—C10	115.8 (2)	C8—C30—C2	108.7 (2)
O1—C9—C8	98.4 (2)	O7—C30—H30	110
C11—C9—C8	113.1 (2)	C8—C30—H30	110
C10—C9—C8	115.2 (2)	C2—C30—H30	110
C1—C10—C9	104.2 (2)	O13—C31—O7	123.5 (3)
C1—C10—C19	110.3 (2)	O13—C31—C32	125.7 (3)
C9—C10—C19	107.8 (2)	O7—C31—C32	110.7 (3)
C1—C10—C5	101.1 (2)	C31—C32—H32A	109.5
C9—C10—C5	122.7 (2)	C31—C32—H32B	109.5
C19—C10—C5	110.0 (2)	H32A—C32—H32B	109.5
C9—C11—C12	111.3 (2)	C31—C32—H32C	109.5
C9—C11—H11A	109.4	H32A—C32—H32C	109.5
C12—C11—H11A	109.4	H32B—C32—H32C	109.5
C9—C11—H11B	109.4	O14—C33—O6	125.3 (3)
C12—C11—H11B	109.4	O14—C33—C34	125.4 (3)
H11A—C11—H11B	108	O6—C33—C34	109.3 (3)
C11—C12—C13	111.5 (2)	C33—C34—H34A	109.5
C11—C12—H12A	109.3	C33—C34—H34B	109.5
C13—C12—H12A	109.3	H34A—C34—H34B	109.5
C11—C12—H12B	109.3	C33—C34—H34C	109.5
C13—C12—H12B	109.3	H34A—C34—H34C	109.5
H12A—C12—H12B	108	H34B—C34—H34C	109.5
C18—C13—C14	108.4 (2)	O16—C35—H35A	109.5
C18—C13—C17	111.0 (2)	O16—C35—H35B	109.5
C14—C13—C17	110.4 (2)	H35A—C35—H35B	109.5
C18—C13—C12	109.9 (2)	O16—C35—H35C	109.5
C14—C13—C12	109.3 (2)	H35A—C35—H35C	109.5
C17—C13—C12	107.9 (2)	H35B—C35—H35C	109.5
C15—C14—C13	112.0 (2)	O16—C35—H35D	109.5
C15—C14—C8	115.8 (2)	H35A—C35—H35D	141.1
C13—C14—C8	115.0 (2)	H35B—C35—H35D	56.3
C15—C14—H14	104.1	H35C—C35—H35D	56.3
C13—C14—H14	104.1	O16—C35—H35E	109.5
C8—C14—H14	104.1	H35A—C35—H35E	56.3
C16—C15—C14	117.7 (3)	H35B—C35—H35E	141.1
C16—C15—H15A	107.9	H35C—C35—H35E	56.3
C14—C15—H15A	107.9	H35D—C35—H35E	109.5
C16—C15—H15B	107.9	O16—C35—H35F	109.5
C14—C15—H15B	107.9	H35A—C35—H35F	56.3
H15A—C15—H15B	107.2	H35B—C35—H35F	56.3
O9—C16—O8	117.8 (3)	H35C—C35—H35F	141.1
O9—C16—C15	122.1 (3)	H35D—C35—H35F	109.5
O8—C16—C15	119.9 (3)	H35E—C35—H35F	109.5
O8—C17—C20	104.9 (2)	C1'—O1—C9	103.5 (2)
O8—C17—C13	113.3 (2)	C1'—O2—C8	106.8 (2)
C20—C17—C13	114.4 (3)	C1'—O3—C1	112.6 (2)
O8—C17—H17	108	C24—O4—C3	119.2 (2)
C20—C17—H17	108	C33—O6—C2	121.0 (2)
C13—C17—H17	108	C31—O7—C30	118.7 (2)
C13—C18—H18A	109.5	C16—O8—C17	122.7 (3)
C13—C18—H18B	109.5	C23—O10—C21	106.0 (3)
H18A—C18—H18B	109.5	C7—O16—C35	116.7 (3)
			
O3—C1—C2—O6	−36.7 (3)	O2—C8—C14—C13	173.8 (2)
C29—C1—C2—O6	91.0 (2)	C30—C8—C14—C13	−67.7 (3)
C10—C1—C2—O6	−161.8 (2)	C9—C8—C14—C13	63.3 (3)
O3—C1—C2—C3	−153.8 (2)	C13—C14—C15—C16	33.8 (4)
C29—C1—C2—C3	−26.1 (2)	C8—C14—C15—C16	−100.8 (3)
C10—C1—C2—C3	81.1 (2)	C14—C15—C16—O9	168.1 (3)
O3—C1—C2—C30	83.7 (3)	C14—C15—C16—O8	−16.5 (4)
C29—C1—C2—C30	−148.7 (2)	C18—C13—C17—O8	−69.8 (3)
C10—C1—C2—C30	−41.4 (3)	C14—C13—C17—O8	50.4 (3)
O6—C2—C3—O4	117.4 (2)	C12—C13—C17—O8	169.7 (2)
C30—C2—C3—O4	−10.7 (3)	C18—C13—C17—C20	50.4 (4)
C1—C2—C3—O4	−133.1 (2)	C14—C13—C17—C20	170.6 (3)
O6—C2—C3—C4	−122.9 (2)	C12—C13—C17—C20	−70.1 (3)
C30—C2—C3—C4	109.0 (2)	O8—C17—C20—C21	−146.0 (3)
C1—C2—C3—C4	−13.4 (2)	C13—C17—C20—C21	89.2 (4)
O4—C3—C4—C28	−69.5 (3)	O8—C17—C20—C22	34.1 (5)
C2—C3—C4—C28	170.2 (2)	C13—C17—C20—C22	−90.7 (4)
O4—C3—C4—C29	167.7 (2)	C22—C20—C21—O10	0.4 (4)
C2—C3—C4—C29	47.4 (2)	C17—C20—C21—O10	−179.6 (3)
O4—C3—C4—C5	58.9 (3)	C21—C20—C22—C23	−0.3 (5)
C2—C3—C4—C5	−61.4 (2)	C17—C20—C22—C23	179.6 (4)
C28—C4—C5—C6	−22.4 (4)	C20—C22—C23—O10	0.2 (6)
C3—C4—C5—C6	−148.3 (2)	O3—C1—C29—C4	−179.6 (2)
C29—C4—C5—C6	109.9 (3)	C10—C1—C29—C4	−57.7 (2)
C28—C4—C5—C10	−148.0 (2)	C2—C1—C29—C4	54.7 (2)
C3—C4—C5—C10	86.1 (2)	C28—C4—C29—C1	177.0 (2)
C29—C4—C5—C10	−15.7 (3)	C3—C4—C29—C1	−62.7 (2)
C10—C5—C6—C7	−158.4 (3)	C5—C4—C29—C1	44.3 (2)
C4—C5—C6—C7	83.2 (3)	O2—C8—C30—O7	64.8 (3)
C5—C6—C7—O15	14.5 (5)	C14—C8—C30—O7	−53.9 (3)
C5—C6—C7—O16	−166.8 (3)	C9—C8—C30—O7	177.3 (2)
O2—C8—C9—O1	−22.2 (2)	O2—C8—C30—C2	−54.8 (3)
C30—C8—C9—O1	−135.4 (2)	C14—C8—C30—C2	−173.5 (2)
C14—C8—C9—O1	89.3 (2)	C9—C8—C30—C2	57.8 (3)
O2—C8—C9—C11	−137.7 (2)	O6—C2—C30—O7	−25.9 (3)
C30—C8—C9—C11	109.1 (3)	C3—C2—C30—O7	103.2 (2)
C14—C8—C9—C11	−26.2 (3)	C1—C2—C30—O7	−141.5 (2)
O2—C8—C9—C10	86.0 (3)	O6—C2—C30—C8	92.3 (3)
C30—C8—C9—C10	−27.2 (3)	C3—C2—C30—C8	−138.6 (2)
C14—C8—C9—C10	−162.5 (2)	C1—C2—C30—C8	−23.3 (3)
O3—C1—C10—C9	−55.7 (3)	O2—C1'—O1—C9	−49.3 (3)
C29—C1—C10—C9	178.3 (2)	O3—C1'—O1—C9	69.7 (3)
C2—C1—C10—C9	71.1 (3)	C2'—C1'—O1—C9	−169.0 (2)
O3—C1—C10—C19	59.7 (3)	C11—C9—O1—C1'	160.9 (2)
C29—C1—C10—C19	−66.3 (3)	C10—C9—O1—C1'	−75.6 (2)
C2—C1—C10—C19	−173.5 (2)	C8—C9—O1—C1'	42.6 (2)
O3—C1—C10—C5	176.1 (2)	O1—C1'—O2—C8	33.2 (3)
C29—C1—C10—C5	50.1 (2)	O3—C1'—O2—C8	−87.3 (3)
C2—C1—C10—C5	−57.0 (3)	C2'—C1'—O2—C8	152.8 (2)
O1—C9—C10—C1	68.8 (2)	C30—C8—O2—C1'	112.8 (2)
C11—C9—C10—C1	−172.0 (2)	C14—C8—O2—C1'	−118.8 (2)
C8—C9—C10—C1	−36.9 (3)	C9—C8—O2—C1'	−5.7 (3)
O1—C9—C10—C19	−48.3 (3)	O2—C1'—O3—C1	59.5 (3)
C11—C9—C10—C19	70.8 (3)	O1—C1'—O3—C1	−55.9 (3)
C8—C9—C10—C19	−154.0 (2)	C2'—C1'—O3—C1	−178.8 (2)
O1—C9—C10—C5	−177.6 (2)	C29—C1—O3—C1'	165.6 (2)
C11—C9—C10—C5	−58.5 (3)	C10—C1—O3—C1'	48.6 (3)
C8—C9—C10—C5	76.6 (3)	C2—C1—O3—C1'	−75.1 (3)
C6—C5—C10—C1	−145.5 (2)	O11—C24—O4—C3	6.9 (4)
C4—C5—C10—C1	−19.6 (2)	C25—C24—O4—C3	−171.5 (2)
C6—C5—C10—C9	99.4 (3)	C4—C3—O4—C24	102.6 (3)
C4—C5—C10—C9	−134.7 (2)	C2—C3—O4—C24	−142.4 (2)
C6—C5—C10—C19	−28.9 (3)	O14—C33—O6—C2	1.8 (4)
C4—C5—C10—C19	97.0 (2)	C34—C33—O6—C2	−178.2 (3)
O1—C9—C11—C12	−142.6 (2)	C3—C2—O6—C33	−50.3 (3)
C10—C9—C11—C12	102.2 (3)	C30—C2—O6—C33	79.2 (3)
C8—C9—C11—C12	−33.9 (3)	C1—C2—O6—C33	−158.8 (2)
C9—C11—C12—C13	66.1 (3)	O13—C31—O7—C30	−11.3 (4)
C11—C12—C13—C18	88.7 (3)	C32—C31—O7—C30	171.0 (3)
C11—C12—C13—C14	−30.1 (3)	C8—C30—O7—C31	137.3 (2)
C11—C12—C13—C17	−150.2 (2)	C2—C30—O7—C31	−104.1 (3)
C18—C13—C14—C15	71.8 (3)	O9—C16—O8—C17	−167.2 (3)
C17—C13—C14—C15	−49.9 (3)	C15—C16—O8—C17	17.2 (5)
C12—C13—C14—C15	−168.4 (2)	C20—C17—O8—C16	−160.4 (3)
C18—C13—C14—C8	−153.2 (2)	C13—C17—O8—C16	−35.0 (4)
C17—C13—C14—C8	85.1 (3)	C22—C23—O10—C21	0.0 (6)
C12—C13—C14—C8	−33.4 (3)	C20—C21—O10—C23	−0.3 (4)
O2—C8—C14—C15	−52.9 (3)	O15—C7—O16—C35	1.1 (6)
C30—C8—C14—C15	65.6 (3)	C6—C7—O16—C35	−177.7 (4)
C9—C8—C14—C15	−163.5 (2)		

### Hydrogen-bond geometry (Å, °)

**Table:**

*D*—H···*A*	*D*—H	H···*A*	*D*···*A*	*D*—H···*A*
C6—H6A···O11^i^	0.97	2.41	3.326 (3)	157
C25—H25B···O1^ii^	0.96	2.34	3.294 (3)	176
C29—H29A···O10^iii^	0.97	2.51	3.348 (3)	145
C34—H34A···O11^iv^	0.96	2.62	3.293 (3)	127

Symmetry codes: (i) *x*−*y*+1, *x*, *z*+1/6; (ii) *x*−*y*, *x*−1, *z*+1/6; (iii) −*x*+2, −*y*+1, *z*−1/2; (iv) −*x*+*y*+1, −*x*+1, *z*−1/3.
